# Waste NR Latex Based-Precursors as Carbon Source for CNTs Eco-Fabrications

**DOI:** 10.3390/polym13193409

**Published:** 2021-10-04

**Authors:** Mohd Adib Hazan, Kar Fei Chan, Khairun Afiqa Jofri, Md Shuhazlly Mamat, Nor Azam Endot, Shahira Liza, Ismayadi Ismail, Mohd Zobir Hussein, Masaki Tanemura, Yazid Yaakob

**Affiliations:** 1 Department of Physics, Faculty of Science, Universiti Putra Malaysia, Serdang 43400, Malaysia; adib.hazan@gmail.com (M.A.H.); kfeichan08@gmail.com (K.F.C.); khairunafiqa@gmail.com (K.A.J.); shuhazlly@upm.edu.my (M.S.M.); 2 Department of Chemistry, Faculty of Science, Universiti Putra Malaysia, Serdang 43400, Malaysia; e_norazam@upm.edu.my; 3 TriPrem i-Kohza, Malaysia-Japan International Institute of Technology, Universiti Teknologi Malaysia, Kuala Lumpur 54100, Malaysia; shahiraliza@utm.my; 4 Materials Synthesis and Characterization Laboratory, Institute of Advanced Technology, Universiti Putra Malaysia, Serdang 43400, Malaysia; ismayadi@upm.edu.my (I.I.); mzobir@upm.edu.my (M.Z.H.); 5 Department of Physical Science and Engineering, Graduate School of Engineering, Nagoya Institute of Technology, Gokiso-cho, Showa-ku, Nagoya 466-8555, Japan; 6 Microscopy Unit, Institute of Bioscience, Universiti Putra Malaysia, Serdang 43400, Malaysia

**Keywords:** waste latex, natural rubber, carbon nanotubes, fractionation, chemical vapor deposition

## Abstract

In this work, the potential of utilizing a waste latex-based precursor (i.e., natural rubber glove (NRG)) as a carbon source for carbon nanotube (CNT) fabrication via chemical vapor deposition has been demonstrated. Gas chromatography-mass spectroscopy (GC-MS) analysis reveals that the separation of the lightweight hydrocarbon chain from the heavier long chain differs in hydrocarbon contents in the NRG fraction (NRG-L). Both solid NRG (NRG-S) and NRG-L samples contain >63% carbon, <0.6% sulfur and <0.08% nitrogen content, respectively, as per carbon-nitrogen-sulfur (CNS) analysis. Growth of CNTs on the samples was confirmed by Raman spectra, SEM and TEM images, whereby it was shown that NRG-S is better than NRG-L in terms of synthesized CNTs yield percentage with similar quality. The optimum vaporization and reaction temperatures were 350 and 800 °C, respectively, considering the balance of good yield percentage (26.7%) and quality of CNTs (I_D_/I_G_ = 0.84 ± 0.08, diameter ≈ 122 nm) produced. Thus, utilization of waste NRG as a candidate for carbon feedstock to produce value-added CNTs products could be a significant approach for eco-technology.

## 1. Introduction

Waste management of latex, either natural or synthetic rubber, has become a serious issue in many countries, as most of the daily and technological applications of latex have a limited lifetime, such as connector rubber rings, rubber cork/stoppers, tires, shoe soles, and so on. The use of natural rubber (NR) products such as laboratory gloves and medical gloves also have increased rapidly due to the advancement of the research sector, as well as due to the awareness of cleanliness and viral diseases, such as with the recent COVID-19 pandemic crisis [1]. The large portion of waste produce from these industry production lines, as well as from post-consumers, will increase rapidly without proper solutions for poor disposal practices, such as land fill dumping and open burning, which expand the financial and environmental burden [2,3]. For example, disposal of rubber gloves will be degraded into micro-plastics that cause possible harm to the health of animals and humans who accidentally ingest them, as they contain heavy metal and organic chemicals. Micro-plastics also have contaminant absorptive properties that may act as pollutants in the environment (air, soil and water) [4]. According to Nuzaimah et al., Malaysia is one of the biggest producers and exporters of these latex products, with 372.4 thousand tons of production and 381.9 thousand tons of consumption for natural rubber in 2020, as mentioned in the Malaysian Rubber Export Promotion Council (MREPC) report. As for world total consumption, an increment of 2.8% per annum until 2025 is forecasted by the International Rubber Study Group (IRSG) [5]. Therefore, development of methods for utilizing these waste matters into value-added products would benefit both the economy and the environment towards eco-technology.

The conventional treatment of waste latex is by pyrolysis, either thermal or catalytic pyrolysis. Both methods have been employed to recycle and upcycle the waste latex by decomposing them into several useful products, such as carbon black, oil, char, fuel and gas [6,7,8,9]. Pyrolysis for waste rubbers has been studied by many researchers, who have shown the compound mainly consists of hydrocarbon compounds (i.e., poly-isoprene (C_5_H_8_)_n_) [10,11,12,13]. Campuzano et al. recently reported distillation of pyrolysis oil from waste tires. They confirmed that the most significant molecular classes found in the fractions were pure hydrocarbons and hydrocarbons containing impurities of one sulfur atom [14]. They also revealed the existence of light aromatic hydrocarbons such as benzene, toluene and xylene, as well as aliphatic-like limonene in those compounds. These findings eventually led to the potential production of a hydrocarbon source from waste NR latex.

Utilizing waste resources, such as agro-waste and waste-plastic, as carbon feedstock has become popular in the promotion of the waste-to-wealth concept [15,16,17,18]. Aligned with this idea, potential value-added products from the upcycling of waste latex as hydrocarbon sources, such as scrap rubber, scrap tire and gloves, are carbon nanostructures (i.e., carbon nanotubes (CNTs) and graphene) [19,20,21,22]. Fabrications of CNTs and graphene have been rapidly gaining attention for various potentials in photonic, electronic, energy and mechanical applications, as they exhibit excellent mechanical, electrical and chemical properties [23,24]. As a case in point, multi-walled CNTs can be utilized as fillers in the development of ceramics and polymer composites, with enhanced optical and mechanical properties [25,26]. Furthermore, multi-walled CNTs also have been employed for scanning probes on micro-scope tips, bio-sensors and field emission devices in industrial applications [27]. There are several techniques that have been developed to produce carbon nanomaterials from cheap, recyclable carbon sources (organic materials, plastic, etc.), such as pyrolysis, plasma treatment, arc-discharge and high-temperature spinning disc processing, depending on the type of precursors and targeted products [28,29]. For CNT fabrication, chemical vapor deposition (CVD) is the most commonly employed method due to its low cost and the uncomplex setup of the system, whereas all types of precursors (i.e., solid, liquid and gaseous) can be exploited. Different vaporization temperatures required for decomposition depending on the precursors type play an important role in determining the growth of desired carbon nanostructures [30]. Therefore, by exploiting the decomposition of hydrocarbon from waste latex, the possibility of its employment as an efficient carbon feedstock for CNTs growth is worth exploring. In this work, we will tackle the synthesis of CNTs by utilizing waste NR gloves, as an economic carbon source, via facile chemical vapor deposition methods. The fabricated CNT product will then be characterized by Raman spectroscopy, scanning electron microscopy and transmission electron microscopy.

## 2. Materials and Methods

### 2.1. Materials

**Figure 1:**
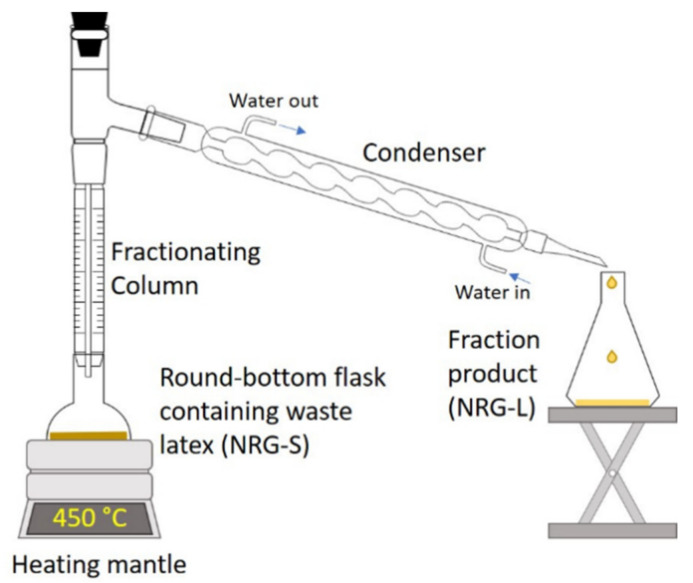
Fractional distillation setup for waste NRG fractionation process.

Waste NR latex employed for this study were commercial NR gloves (NRG) from laboratory waste (AS ONE Corporation, 1-8449-01, Made in Malaysia). Two different types of precursors were prepared, which were solid (NRG-S) and liquid-fraction (NRG-L). For the NRG-fraction process, 50 g of NRG-S placed inside a 1 L round-bottom flask was first heated up to 120 °C for 2 h to remove the water content from the waste. Then, the fraction distillation setup was prepared in the fume hood, as shown in Figure 1. The NRG-S was heated above the boiling point of 450 °C until there was only residue left in the flask. NRG-S vapor was condensed in the condenser column then moved into the collector beaker. The fraction (NRG-L) was then analyzed for comparison.

### 2.2. Precursor Analysis

The elemental composition of the gloves was determined using the Carbon-Nitrogen-Sulphur analyser (CNS, Version 1.1x, TruMac, LECO Corporation, MI, USA) to determine the carbon, nitrogen and sulfur contents. Thermal gravimetric analysis (TGA, TGA/ SDTA 851, Mettler-Toledo Inc., Greifensee, Switzerland) was performed to determine the decomposition temperature. The analysis was done under nitrogen condition, heated up to 800 °C. To investigate the precursors hydrocarbon contents, the waste NRG was injected into a gas chromatography-mass spectroscopy system (GC-MS, 8060 MS with Cryo 800 module, Fisons Plc., Loughborough, UK) in the EI (Electron Impact) mode, with the electron energy set at 70 eV and the mass range at m/z 25–700. The chromatographic separation was performed using a capillary column ZB-5 MS (30 m × 0.25 mm, (length × internal diameter)), at 0.25 µm film thickness. The temperature program was set as follows: Temperature of 50 to 100 °C with a step increment of 20 °C/min, 1 min holding time; 100 to 300 °C with step increment of 5 °C/min, then 10 min holding time. The splitless injection with a split ratio of 2:1 was applied at 290 °C. The ion source and transfer line temperatures were kept at 240 and 320 °C, respectively. The flow rate of the carrier gas (helium) was maintained at 1.0 mL/min. The chromatographic peaks were recognized using their retention times by comparing the retention times with those of authentic compounds, and the spectral data were obtained from the Wiley NIST Spectral Libraries. The peak identification was carried out by probability-based matching (PBM).

### 2.3. Fabrication of CNTs

**Figure 2:**
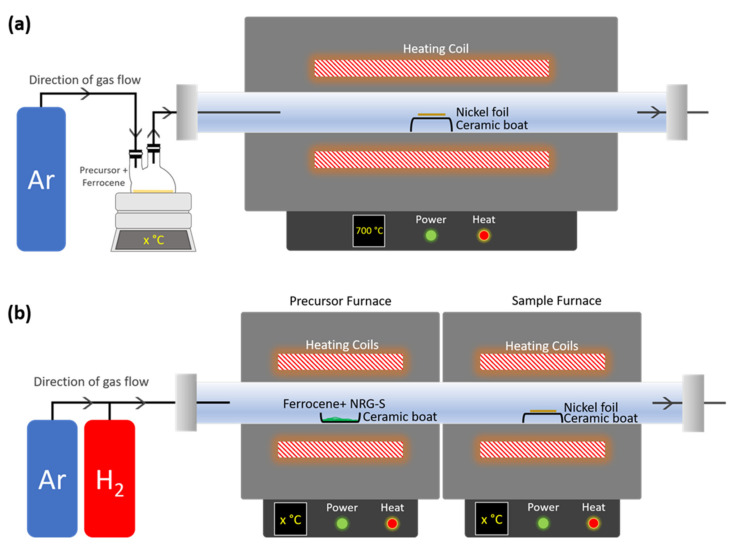
Chemical vapor deposition setup for CNTs synthesis process. (**a**) Single furnace CVD for phase 1 and (**b**) double furnace CVD for phase 2.

The synthesis of CNTs was conducted in two phases by using two different CVD techniques. For phase 1, investigation of the potential of NRG-S and NRG-L as carbon precursors was performed using CVD with a single furnace setup, as shown Figure 2a. The commonly used precursor (i.e., ethanol) was employed for CNTs product comparison, as liquid precursors have a lower cost and are easier to handle than gaseous precursors in our CVD system [30]. A total of 5 wt% of ferrocene was mixed with the precursor in the heating flask as catalyst nanoparticle, which assisted as seed for CNTs growth, while nickel foil (2 cm × 2 cm) was placed in the ceramic boat at the center of tube furnace (cylindrical quartz tube, length = 100 cm and diameter = 5 cm) as a substrate. Before the start of the CNTs synthesis process, Ar gas was introduced at 150 sccm into the tube while increasing the furnace temperature to 700 °C, whereby the synthesis reaction occurred at this temperature [31]. When the furnace reached the reaction temperature, the precursor was vaporized above the boiling point using heating mantle at 450 °C for NRG and 80 °C for ethanol, respectively. The carbon feedstock was injected into the tube reactor by by-passing the Ar gas flow for 30 min. Afterwards, the samples were left to cool by switching off the furnace power until room temperature was reached, while maintaining the Ar environment. The synthesized samples were then collected from the nickel substrate.

For phase 2, investigation of the effect of NRG-S precursor vaporization temperature and sample reaction temperature on CNTs growth was performed via double furnace CVD (DFCVD), as in Figure 2b. A total of 5 wt% of ferrocene was mixed with the precursor (0.4 g) in the ceramic boat at the ‘precursor furnace’, while nickel foil (1 cm × 1 cm), as a substrate, was placed in the ceramic boat at the ‘sample furnace’ (cylindrical quartz tube, length = 90 cm and diameter = 4 cm). Before increasing the temperature, Ar:H_2_ gas flow with 90:10 sccm were introduced in the reactor for 10 min. Then, the ‘sample furnace’ temperature was increased until it reached the desired reaction temperature. For this study, the sample reaction temperature performed at 700–900 °C. After that, the ‘precursor furnace’ temperature was increased to the studied vaporization temperature of 350–450 °C. Upon reaching the studied temperature, the synthesis processes were done for 15 min. Afterwards, the samples were left to cool by switching off the furnace power until room temperature was reached, while maintaining the Ar environment. The synthesized samples were then collected from the nickel substrate.

### 2.4. Characterizations

For CNTs yield analysis, the weights of the catalyst and synthesized samples were measured to calculate the carbon deposition yield percentage [32]:

(1) $$\mathrm{Yield}=\frac{m_{f}-m_{i}}{m_{i}}\times 100$$

where m_i_ denotes the catalyst initial weight and m_f_ is the total weight of the substrate after carbon deposition. A scanning electron microscope (SEM, JSM 5600, JEOL Ltd. Tokyo, Japan; accelerating voltage: 15 kV) was employed for the morphology observation of the samples surface. Raman spectroscopy (NRS 3300 laser Raman spectrometer, JASCO Inc., Tokyo, Japan; laser excitation energy = 532.08 nm, spectral grating = 600 L/mm; laser power = 7.8 mW; integration time per spectra = 15 s; accumulation per spectrum = 3x) was used to analyze the quality of the synthesized CNTs. Additionally, 4 more spectra were measured per sample in order to complete the average I_D_/I_G_ ratio calculation. Fourier transform infrared spectroscopy (FTIR, FT/IR-4200A, JASCO Inc., Tokyo, Japan; standard light source; triglycine sulphate detector; accumulation per spectrum: 235, aperture: 7.1 mm; scanning speed: 2 mm/s; filter: 30 kHz) was used to characterize the structure and surface property of the samples using the attenuated total reflectance (ATR) technique. High-resolution nano-structural observation and element confirmation of the synthesized CNTs were conducted using scanning transmission electron microscopy (STEM, JEM-ARM200F, JEOL Ltd., Tokyo, Japan; accelerating voltage = 200 kV) equipped with bright field (BF), annular dark field (ADF) and energy dispersive X-ray analysis (EDX) detector.

## 3. Results and Discussions

### 3.1. Analysis of Waste NRG as Carbon Precursor

**Figure 3:**
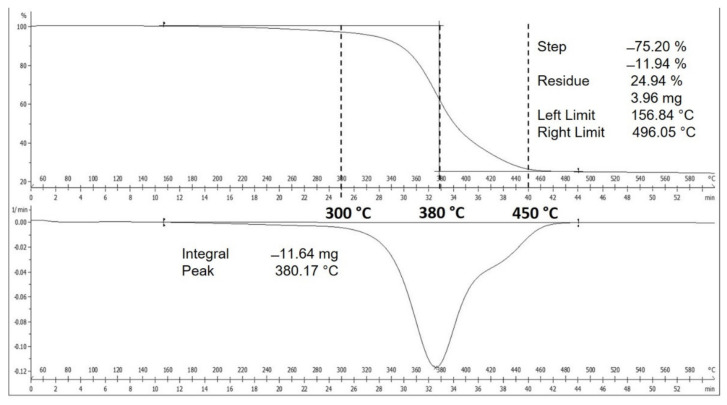
TGA and DTG curves of waste NRG.

In order to explore the potential of NRG as precursors, TGA analysis was performed to identify the temperature where NRG liberated most of its compounds. The thermal gravimetric analysis and differential thermo-gravimetric (TGA-DTG) profile, in Figure 3, shows a single-step weight loss in the temperature range of 170–470 °C, reaching a peak at 380 °C. The weight loss from room temperature up to 200 °C resulted from the removal of water content inside the sample. A further significant weight loss trend was seen at 300–450 °C, which suggested that most of the compounds were liberated in this temperature range. The remaining 24.94% was the carbonaceous residue left after hydrocarbon compound evaporation.

From CNS analysis, the percentages of elements are shown in Table 1. It shows that both the NRG and its fraction contain >63% of high carbon, with only a very low amount of sulfur and nitrogen, being <0.6% and <0.08%, respectively. The remaining percentage might be contributed to by other elements such as oxygen and hydrogen. The majority content percentage being carbon means it will comply as a potential carbon source, while the small amount of sulfur and nitrogen will assist the growth of CNTs [33,34].

**Figure 4:**
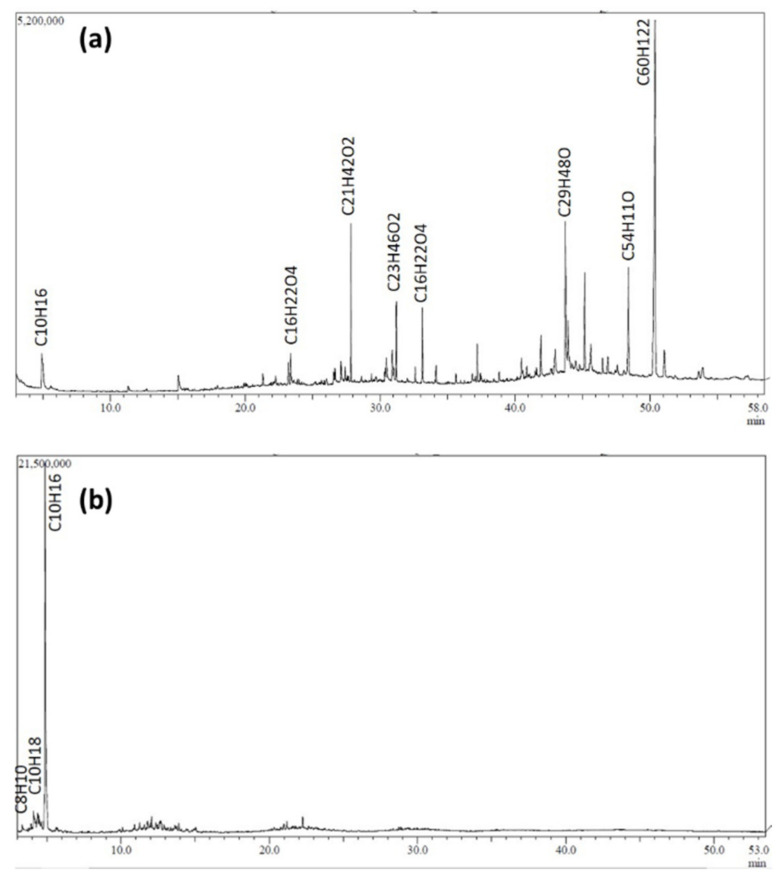
GC-MS chromatogram profile of waste NRG and its fraction. (**a**) NRG-S and (**b**) NRG-L.

GC–MS analysis was performed to identify the waste NRG compounds. The chromatogram profiles of the waste NRG-S and NRG-L are shown in Figure 4a,b, respectively. Both of the chromatograms show the presence of a noise peak, thus the peaks from the wastes were counted if the peak matched 80% to specific compounds with high intensity. The peak identification was carried out by probability-based matching (PBM) [35]. The major compounds in the NRG are tabulated in Table 2 and listed in order of the corresponding peaks in each GC spectrum. The results revealed that NRG-S contains more carbon elements of heavy hydrocarbon compounds ranging from C_16_ to C_29_, compared to NRG-L with C_8_ to C_10_. This is due to the fractionation process, whereby the NRG-L was reacted with an oxygen element in the samples and the environment before releasing the carbon component in the form of CO_2_, CO or another light hydrocarbon compound into the surrounding [36,37]. From the chromatogram profile, the compounds inside the NRG-S mostly cracked after the 20 min retention time of heating process, which was at 170 °C, due to the presence of high hydrocarbon molecular weight components. Whereas for NRG-L the compound started to vaporize early, at 3 min retention time, due to its lower molecular weight components. The presence of compounds such as limonene and dimethyl benzene is in agreement with the literature [13]. The long hydrocarbon chains (i.e., aliphatic and aromatic) were derived from the additive and thermal degradation process [38]. Branched aliphatic hydrocarbon- and benzene-based compounds with high molecular weight are suspected as the major hydrocarbon structures present in the solid sample, as depicted in the unknown peaks of Figure 4a [23]. Expected compounds were determined as hexacontane, cyclohexylmethyl hexyl ester, di- and trimethyl benzene and aliphatic alcohol. They could be tetramer, pentamer or hexamer, which would appear at a high retention time due to their high molecular weight.

### 3.2. Characterization of Synthesised CNTs

**Figure 5:**
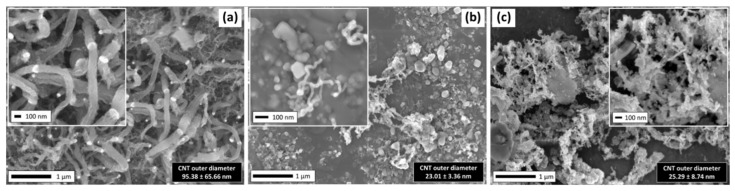
FESEM images of samples synthesized from (**a**) ethanol, (**b**) NRS-L and (**c**) NRG-S precursors.

The investigation of the potential of NRG-S and NRG-L as carbon precursors was performed with ethanol as a comparison precursor. The surface morphology of the three samples were shown in FESEM images (see Figure 5). Figure 5a shows that a high carbon yield percentage of 114.6% and an average CNTs diameter of 95.38 ± 65.66 nm were synthesized from the ethanol precursor. Other than fibrous CNTs structures, flake-like carbon protrusions and debris were also spotted. In comparison, the NRG precursors show a lower yield percentage with smaller carbon nanostructured products, as shown in Figure 5b,c. NRG-S showed better results, with 27.4% carbon yield, compared to NRG-L with only 6.7%, whereas both of their average diameters were approximately 25 nm.

**Figure 6:**
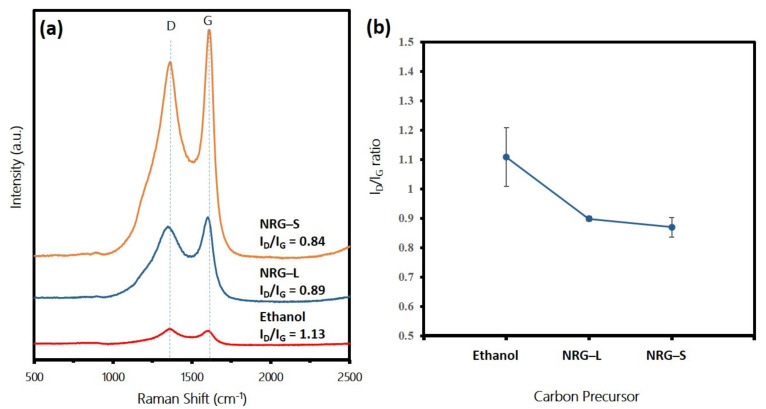
(**a**) Raman spectra of samples synthesized from ethanol, NRS-L and NRG-S precursors. (**b**) Corresponding average I_D_/I_G_ ratio.

Raman spectroscopy was employed to characterize the quality of synthesized CNTs. In general, the Raman peaks used for carbon analysis contained the G peak to indicate the degree of the graphitization and the D peak to indicate the degree of defect in the graphite structure. Raman peaks for carbon analysis containing the G peak (indicating the graphitization) arise from highly ordered sp^2^-bonded carbon materials, and those containing the D peak (indicating the defect in the carbon structure) arises from disordered sp^3^-bonded carbon materials and carbonaceous impurities [39].The Raman spectra in Figure 6a indicate that all the samples produced D and G peaks in the range of 1349.89–1351.53 and 1596.89–1610.53 cm^−1^, respectively, proving the presence of the carbon materials on the samples. The small shift of the peaks and the variance of the intensities indicate the different crystallinity degrees of the CNTs produced. The intensity is proportional to the amount of disorder (crystallite boundary) in the sample. The ratio between the intensities of the defect (D band) and the graphitization (G band), that is, I_D_/I_G_, provides a parameter that can be used for quantifying disorder. The lower I_D_/I_G_ ratio indicating a higher quality synthesis of CNTs [40]. For the sample using ethanol as the precursor, the D peak was higher compared to the G peak, indicating there were more defects compared to graphitization of carbon. In contrast, for NRG-S and NRG-L cases, the G peaks were higher compared to D peaks. The average I_D_/I_G_ ratio of the samples from ethanol, NRG-L and NRG-S were 1.11 ± 0.10, 0.89 ± 0.01 and 0.87 ± 0.03, respectively (see Figure 6b). Samples from ethanol contained a lot of defects and amorphous carbon, which was due to the non-optimized vaporization temperature of the precursor (above boiling temperature of 80 °C), thus resulting in an excessive amount of carbonaceous supply during deposition. For NRG cases, the peak vaporization temperature was high (i.e., 380 °C). Therefore, condensation into liquid form may have occurred along the connection tube, which was at room temperature, before entering the reaction chamber, resulting in a low amount of carbon feedstock for CNTs growth. Higher intensity peaks for NRG-S compared to NRG-L indicate a higher percentage of carbon yield on the samples.

### 3.3. Effect of Precursor Vaporization Temperature on CNTs Growth

**Figure 7:**
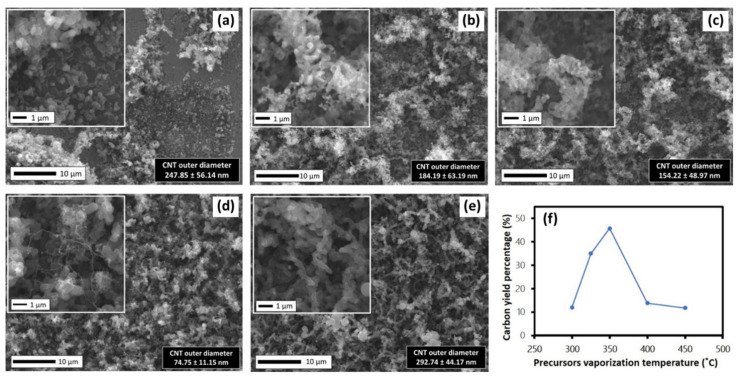
SEM images of samples synthesized at various precursor vaporization temperatures of (**a**) 300, (**b**) 325, (**c**) 350, (**d**) 400 and (**e**) 450 °C, and (**f**) carbon yield percentage.

Even though the TGA analysis shows that the NRG totally decomposed at 450 °C, the previous results in Section 3.1 suggested that the vaporization temperature played an important role in supplying the carbon source for CNTs growth in the reaction chamber. Therefore, a double-furnace CVD was employed to eliminate the NRG-S precursor condensation factor during the synthesis process. The SEM images of the CNTs synthesized at the vaporization temperature of 300–450 °C are shown in Figure 7. Figure 7f shows that the yield percentage trend increased at the vaporization temperature of 300–350 °C, then decreased at 400–450 °C. The results indicate that below the vaporization temperature of 350 °C, insufficient energy produced a low quantity of carbon supply, resulting in a low growth rate of CNTs. Figure 7a–c presents a highly dense, short fibrous structure, where the diameter of CNTs became smaller with increments of temperature. The large diameter of the CNTs was due to large Fe clusters attaching to the substrate, resulting in the poor catalytic effect, as the ferrocene did not fully vaporize at low temperature. On the other hand, at the vaporization temperature of 400 °C and above, an excessive amount of hydrocarbon supply was produced onto the catalyst surface, including hydrocarbon radicals. Hence, the catalyst encapsulated by the carbon particles resulted in the reduction of catalytic activity which hindered the growth of CNTs [41,42]. Figure 7d,e shows chaplet-shaped carbon was dominant on the sample surface, with some of the fibrous structure attached. The larger CNTs diameter was also due to agglomeration of Fe and Ni catalysts into larger particles at high temperature, hence affecting CNTs growth.

**Figure 8:**
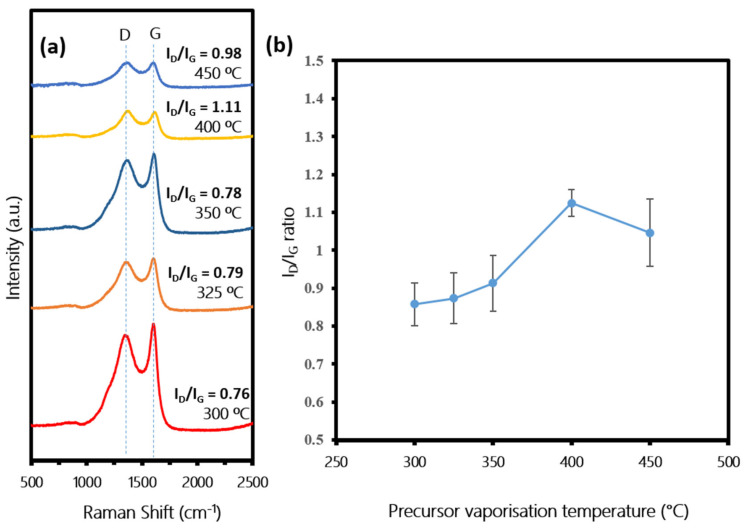
(**a**) Raman spectra of samples synthesized at various precursor vaporization temperatures of 300, 325, 350, 400 and 450 °C. (**b**) Corresponding average I_D_/I_G_ ratios, respectively.

Figure 8a shows the Raman spectra of the fabricated CNTs at different precursor vaporization temperatures. All samples showed the two prominent peaks D and G in the range of 1349.89–1362.53 cm^−1^ and 1597.26–1610.53 cm^−1^, respectively, indicating that the carbon materials successfully diffused on the samples of the CNTs produced with varying crystallinity. A clear pattern of average I_D_/I_G_ ratios were observed in Figure 8b. At low vaporization temperatures of 300–350 °C, the I_D_/I_G_ ratios were in the range of 0.76–0.92, indicating good quality of CNTs crystallinity. As the temperature increased to 400–450 °C, the I_D_/I_G_ ratios increased in the range of 1.09–1.19. These results are in agreement with SEM images in Figure 7, which indicate that, at low vaporization temperature, sufficient energy needs to be supplied to obtain both a percentage of high carbon yield and high quality (small diameter and good crystallinity) CNTs. Contrarily, too high energy of vaporization temperature will result in an excessive amount of hydrocarbon supply, including radical, which will reduce the catalytic activity of the catalyst. This will produce CNTs with a highly disordered structure, amorphous chaplet-shaped carbon and other carbonaceous impurities. One more factor to be considered is that the release of low molecular weight hydrocarbon was dominant at low vaporization temperature compared to high molecular weight hydrocarbon, as per GC-MS results. It is reported that simpler hydrocarbon favors the formation of CNT, hence resulting in a higher yield of CNTs [43,44].

### 3.4. Effect of Sample Reaction Temperature on CNTs Growth

**Figure 9:**
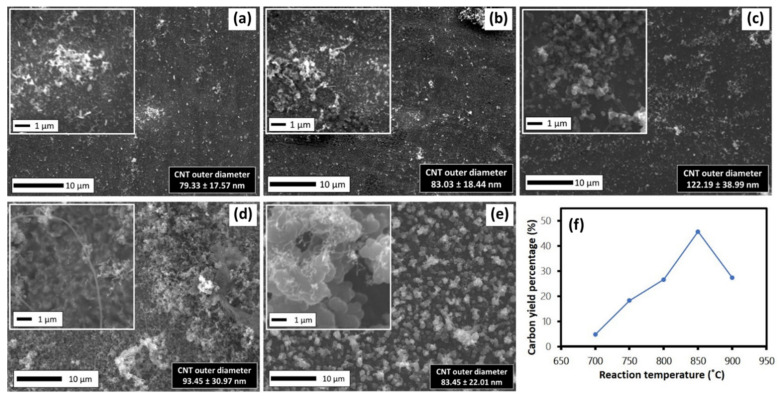
SEM images of samples synthesized at various sample reaction temperatures of (**a**) 700, (**b**) 750, (**c**) 800, (**d**) 850 and (**e**) 900 °C, and (**f**) carbon yield percentage.

To investigate the effect of sample reaction temperature, the vaporization temperature of the precursors was fixed at 350 °C, while the reaction temperature was set in the range of 700–900 °C, with a step increment of 50 °C. Figure 9a–e shows SEM images of the surface morphology of the samples. The carbon yield percentages (see Figure 9f) and diameters of the CNTs showed similar upward trends and then reduced at 900 °C, respectively. In terms of carbon yield percentages, as the temperature increased, it enhanced the ferrocene pyrolysis for fine Fe clusters, producing more nucleation sites on the Ni substrate, which increased the chance for the growth of CNTs. However, the formation of iron carbide particles can easily occur at very high synthesis temperatures, so that can be another factor that reduces the growth of CNTs at 900 °C. In relation to the diameter of CNTs, larger diameters were attributed to by agglomeration of Fe and Ni catalysts. For samples at 850 and 900 °C, even though the diameter of CNTs were in the range of approximately 83–93 nm, they were mixed and attached with the background of large particles and the chaplet-shaped structure, which is believed to be impurities of amorphous carbon.

**Figure 10:**
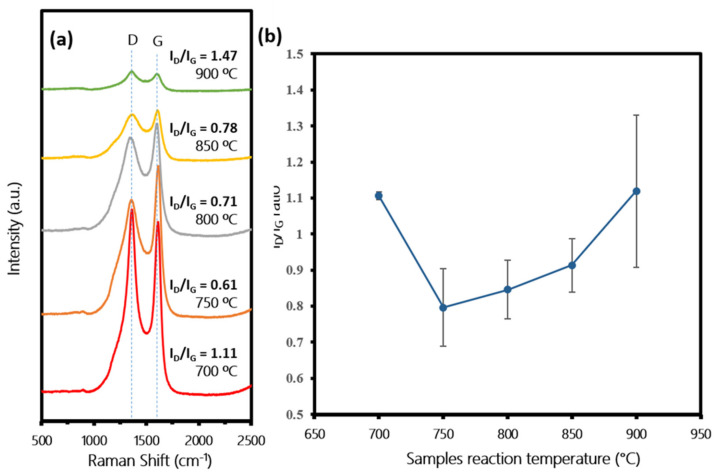
(**a**) Raman spectra of samples synthesized at various sample reaction temperatures of 700, 750, 800, 850 and 900 °C. (**b**) Corresponding average I_D_/I_G_ ratio, respectively.

Figure 10a shows the Raman spectra of the fabricated CNTs samples at different reaction temperatures. All samples showed the two prominent peaks D and G in the range of 1353.53–1361.53 and 1593.89–1613.53 cm^−1^, respectively, indicating that the carbon materials successfully diffused on the samples of CNTs produced with varying crystallinity. A clear pattern of I_D_/I_G_ ratios were observed in Figure 10b. At the low reaction temperature of 700 °C, the average I_D_/I_G_ ratio was 1.11 ± 0.01, indicating the poor crystallinity of CNTs. At the temperature of 750–850 °C, the I_D_/I_G_ ratio was lower, in range of 0.61–0.97, indicating the higher quality of crystallinity of the produced CNTs. However, as the temperature increased to 950 °C, the I_D_/I_G_ ratio increased again up to 1.47 (average I_D_/I_G_ = 1.12 ± 0.21). These results are in agreement with SEM images in Figure 9, which indicate that a too low reaction temperature contributed to low catalyst–precursor activity, while a too high reaction temperature contributed to agglomeration of catalysts and the formation of iron carbide, resulting in the poor quality of the produced CNTs.

**Figure 11:**
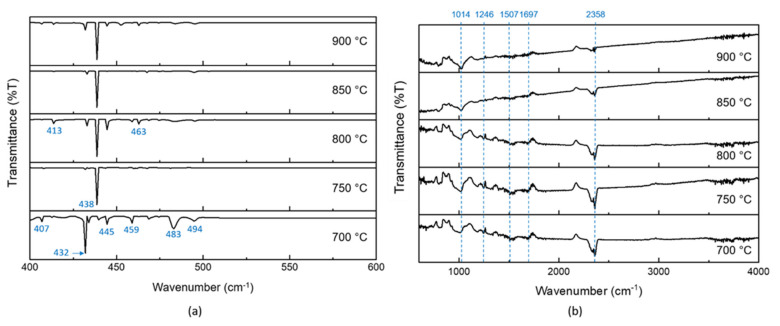
FTIR spectra of samples synthesized at various sample reaction temperatures of 700, 750, 800, 850 and 900 °C. (**a**) The wavenumber ranged from 400 to 600 cm^−1^ and (**b**) the wavenumber from 600 to 4000 cm^−1^.

Figure 11 depicts the FTIR spectra of samples (700–900 °C). In Figure 11 a (wavenumber ranged from 400 to 600 cm^−1^), the FTIR spectra displayed transmission bands at 432 and 455–469 cm^−1^, which corresponded to the Fe-O bonding from the metal catalyst [45,46]. The samples exhibited metal cation lattice modes at 407 cm^−1^ [47], magnetite structure at 483 cm^−1^ [48] and ferrocene structure at 494 cm^−1^ [49]. Additionally, the sulfur component from the precursor (waste glove) contributed to the transmission band at 413 and 438 cm^−1^, with respect to S-S stretching and SO_4_^2-^ [45,50]. The FTIR spectra in Figure 11b, ranging in wavenumber from 600 to 4000 cm^−1^, displayed the carbon component within the samples. The main transmission bands were related to –CONH bonding at 1014 cm^−1^, CH-CH_3_ stretching at 1246 cm^−1^, the C=C bond at 1507 cm^−1^, the C=O bond at 1697 cm^−1^ and the non-conjugate carboxylic group at 2358 cm^−1^ [46,51,52,53]. The transmission band at 1507 cm^−1^ formed due to the graphitic carbon E_1u_ mode which underwent sp^2^ hybridization.

**Figure 12:**
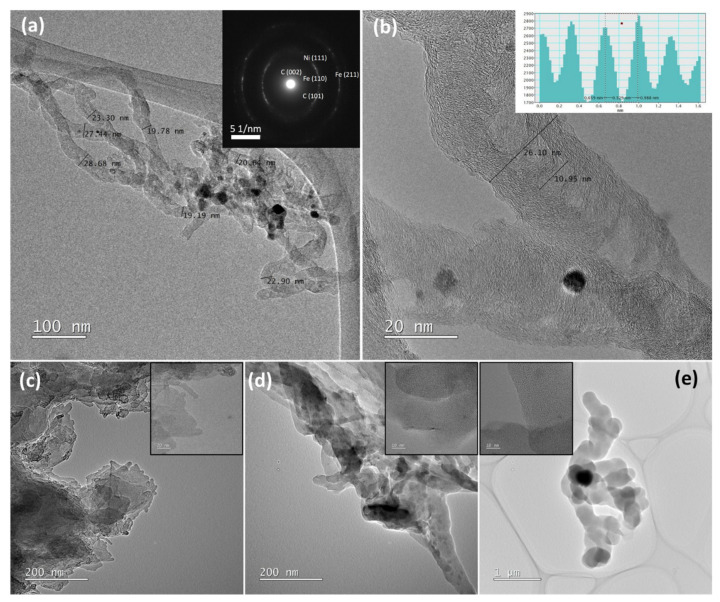
HRTEM images of the samples synthesized at 800 °C. (**a**) Low magnification image of CNTs (inset is corresponding SAED pattern), (**b**) high magnification image of CNTs (inset is corresponding lattice profile), (**c**) bundles of short CNTs, (**d**) amorphous carbon nanofiber and (**e**) chaplet shape of the amorphous carbon.

Figure 12 shows transmission electron microscopy (TEM) observations of the synthesized CNTs at 800 °C. The TEM images in Figure 12a depict an overview of the distribution of the CNTs, with an average diameter of approximately 25 nm. The high magnification image in Figure 12b reveals the graphitic layers of the multi-walled CNTs with inner and outer diameters of 10.95 and 26.10 nm, respectively. The lattice profile and the selected area electron diffraction (SAED) image shown in Figure 12a,b, inset, confirmed the characteristic 0.33 nm inter-wall spacing and (002), (101) ring planes of C that indicate the graphitic layers. Nearby crystallinity of catalysts were also detected as (110), (211) ring planes of Fe and (111) ring planes of Ni. Other shapes of carbon nanostructures are also revealed in Figure 12c–e, such as bundles of short CNTs, the large diameter of amorphous carbon nanofibers with slight graphitization and chaplet shape of the amorphous carbon.

**Figure 13:**
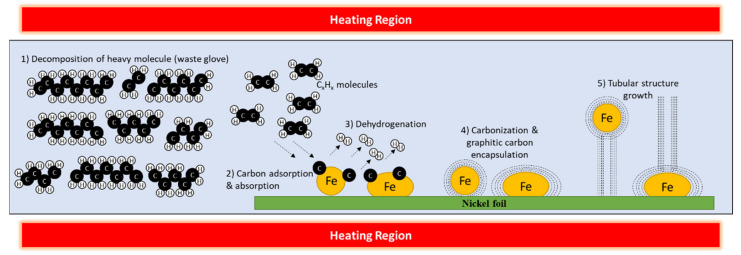
Illustration of the suggested growth mechanism of CNTs during the CVD process.

The growth mechanisms suggested from these finding were by tip growth model or base growth model, depending on the reaction of the nanoparticle catalyst interaction with the substrate [54,55]. There is not enough evidence to clarify the catalyst reaction as it was encapsulated inside CNTs during the growth process. As the NRG-S and ferrocene pyrolyzed to become the hydrocarbon chain and Fe nanoparticles, respectively, the vapor would have been dissolved and adsorbed on the Ni substrates in the reaction chamber. Fe nanoparticles will introduce more nucleation sites on the Ni substrates, while Ni will elevate hydrocarbon decomposition to aid the growth of CNTs [56]. When the vaporized precursor reached the reaction temperature, the hydrocarbon chains were decomposed, and carbon adsorption and dehydrogenation of the hydrocarbon occurred on the catalyst. Lastly, carbonization and graphitization occurred on the catalyst surface. Tubular or fibrous structures would continue to grow with the continual supply of suitable hydrocarbon. The suggested growth mechanism of CNTs is illustrated and summarized in Figure 13.

**Figure 14:**
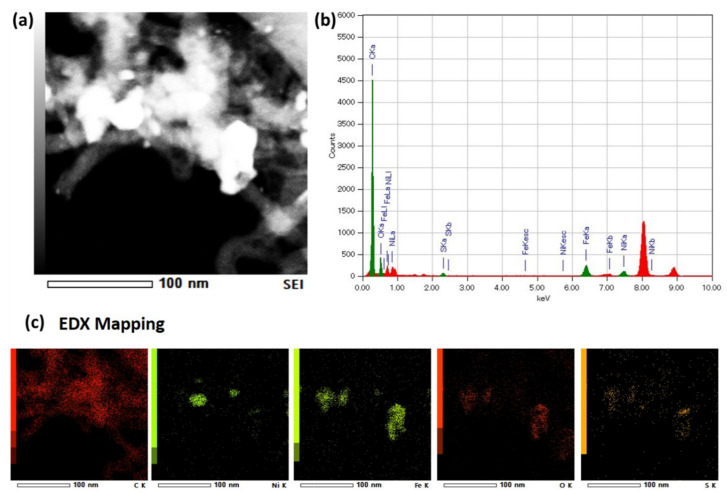
Scanning transmission electron microscopy (STEM) image in the selected area of the CNTs. (**a**) Bright-field image, (**b**) corresponding EDX spectra, and (**c**) EDX element distribution mapping.

The elemental composition was analyzed using energy dispersive spectroscopy (EDS) of samples in Figure 14. Figure 14a shows bright field STEM images of the CNTs, whereby heavy elements as metal catalyst can be observed clearly. Figure 14b,c shows element composition and mapping in the area. High intensity of C indicates the domination of CNTs growth in the samples. Fe, Ni, and O were contributed by the catalyst itself, which might be oxide produced by oxygen in the precursors and the environment. The low presence of sulfur, S, was contributed to by NRG, which aids in improving the growth of CNTs [34,57]. Additionally, Figure 14c shows that Ni and Fe were mixed and agglomerated among the CNTs, indicating both catalysts play a role in assisting the growth process of CNTs. The obtained results suggest that there is the possibility of NRG being a candidate as a carbon precursor for the fabrication of CNTs. Further optimization will need to be carried out for other parameters, such as growth time and catalyst factor that effect the formation of CNTs. These factors will be discussed in our forthcoming papers.

## 4. Conclusions

In this study, we successfully fabricated CNTs using the chemical vapor deposition methods by utilizing waste NRG as carbon precursor. The fractional distillation of NRG-S under an inert environment was performed to produce NRG-L. CNS analysis showed both NRG samples had carbon content higher than 63%, while sulfur and nitrogen contents were lower that 0.6% and 0.08%, respectively. GC-MS analysis revealed the separation of the lightweight hydrocarbon chain from the heavier long chain, with differences in the hydrocarbon contents in the NRG fraction (NRG-L). The morphology and Raman spectra exhibit that NRG-S produces a higher yield of CNTs with similar quality, compared to NRG-L under the same synthesis conditions. The yield percentage of CNTs, diameter and crystallinity were shown to be dependent on synthesis conditions, (i.e., precursor vaporization temperature and sample reaction temperature varied in this study). The results revealed that vaporization of precursors at low temperatures below 350 °C resulted in low carbon yield percentage and low quality of CNTs due to insufficient energy supplied. In contrast, the higher energy of high vaporization temperatures resulted in an excessive amount of hydrocarbon supply, including radical, which reduced the catalytic activity of the catalyst. On the other hand, a too low sample reaction temperature of 700 °C contributed to low catalyst–precursor activity, while a too high reaction temperature of 900 °C contributed to agglomeration of the catalyst and the formation of iron carbide, resulting in the poor quality of the produced CNTs. In this work, the optimum precursor vaporization and sample reaction temperatures were 350 and 800 °C, respectively, considering the balance of good yield percentage (26.7%) and quality of the produced CNTs (I_D_/I_G_ = 0.84 ± 0.08, diameter ≈ 122 nm). Further optimization of the process and oxidative treatment will be explore in our forthcoming work in order to improve the quality, yield and purity of the product for application purposes [27]. Thus, our findings show that NRG can be utilized as a good candidate of a carbon source for CNTs production towards eco-technology.

## Figures and Tables

**Table 1:** Composition of waste NRG from CNS analysis.

Sample	Carbon (%)	Nitrogen (%)	Sulfur (%)
NRG-S	64.858	0.0706	0.6669
NRG-L	63.820	0.0769	0.4791

**Table 2:** Major compounds analyzed from GC-MS for waste NRG-S and its fraction (NRG-L).

Retention Time (Min)	Expected Compound	Formula
**NRG-S**		
4.111	d-Limonene	C_10_H_16_
23.21	Dibutyl phthalate	C_16_H_22_O_4_
27.841	Nonadecyl acetate	C_21_H_42_O_2_
31.206	Heneicosyl acetate	C_23_H_46_O_2_
33.135	1,2-benzenedicarboxylic acid	C_16_H_22_O_4_
43.946	Fucosterol	C_29_H_48_O
48.475	Tetrapentacontane	C_54_H_110_
50.398	Hexacontane	C_60_H_122_
**NRG-L**		
3.343	Benzene, 1,3-dimethyl-	C_8_H_10_
4.111	d-Limonene	C_10_H_16_
4.35	2,6-Dimethyl-2-trans-6-octadiene	C_10_H_18_
4.479	1,5-Heptadiene,2,3,6-trimethyl-	C_10_H_18_
4.896	d-Limonene	C_10_H_16_

## Data Availability

The data presented in this study are available on request from the corresponding author.
